# Application of TonB-Dependent Transporters in Vaccine Development of Gram-Negative Bacteria

**DOI:** 10.3389/fcimb.2020.589115

**Published:** 2021-01-27

**Authors:** Jia Wang, Kun Xiong, Qu Pan, Weifeng He, Yanguang Cong

**Affiliations:** ^1^ Department of Clinical Laboratory, Traditional Medicine Hospital Affiliated to Southwest Medical University, Luzhou, China; ^2^ Department of Cold Environmental Medicine, Institute of High Altitude Military Medicine, Army Medical University, Chongqiong, China; ^3^ Department of Microbiology, Chengdu Medical College, Chengdu, China; ^4^ Department of Burn, Southwest Hospital, Army Medical University, Chongqing, China; ^5^ Precision Medicine Center, Traditional Medicine Hospital Affiliated to Southwest Medical University, Luzhou, China

**Keywords:** TonB-dependent transporter, vaccine, infection, Gram-negative bacteria, immune

## Abstract

Multiple scarce nutrients, such as iron and nickel, are essential for bacterial growth. Gram-negative bacteria secrete chelators to bind these nutrients from the environment competitively. The transport of the resulting complexes into bacterial cells is mediated by TonB-dependent transporters (TBDTs) located at the outer membrane in Gram-negative bacteria. The characteristics of TBDTs, including surface exposure, protective immunogenicity, wide distribution, inducible expression *in vivo*, and essential roles in pathogenicity, make them excellent candidates for vaccine development. The possible application of a large number of TBDTs in immune control of the corresponding pathogens has been recently investigated. This paper summarizes the latest progresses and current major issues in the application.

## Introduction

The peptidoglycan of Gram-negative bacteria is encompassed with an outer membrane layer, which serves as a selective permeation barrier and protects bacterial cells from the damage of harmful compounds in the environment. Small hydrophilic nutrients of < 600 daltons can cross the outer membrane into the periplasmic space *via* porins in a passive diffusion manner (Nikaido, 2003; Schalk et al., 2012). However, some essential nutrients with large molecular weight or as components of large compounds, which are substantially large to pass through the porin channels, are transported by utilizing TonB-dependent transporters (TBDTs, or TonB-dependent receptors) in the outer membrane (Krewulak and Vogel, 2011).

Most characterized TBDTs are involved in iron uptake (Stork et al., 2013). In vertebrate hosts, iron is sequestered into host proteins, including hemoglobin, myoglobin, transferrin, and ferritin. The iron sequestration is an important defense mechanism that limits the growth of invading pathogens, a strategy known as “nutritional immunity” (Hennigar and McClung, 2016). Pathogens secrete siderophores to bind and chelate iron from iron-containing proteins to circumvent nutritional immunity. Siderophore-iron complexes are subsequently transported into the periplasmic space *via* TBDTs in an energy-requiring process (Hennigar and McClung, 2016).

TBDTs are excellent candidates for vaccine development due to their critical roles in bacterial virulence and their surface exposure feature. Thus far, numerous TBDTs have been demonstrated to have vaccine potential against various pathogens. In the present paper, the latest advancements in the application of TBDTs in the vaccine development of Gram-negative bacteria are reviewed, and their future in vaccine application is explored.

## Structure and Function of TBDT

**Figure 1:**
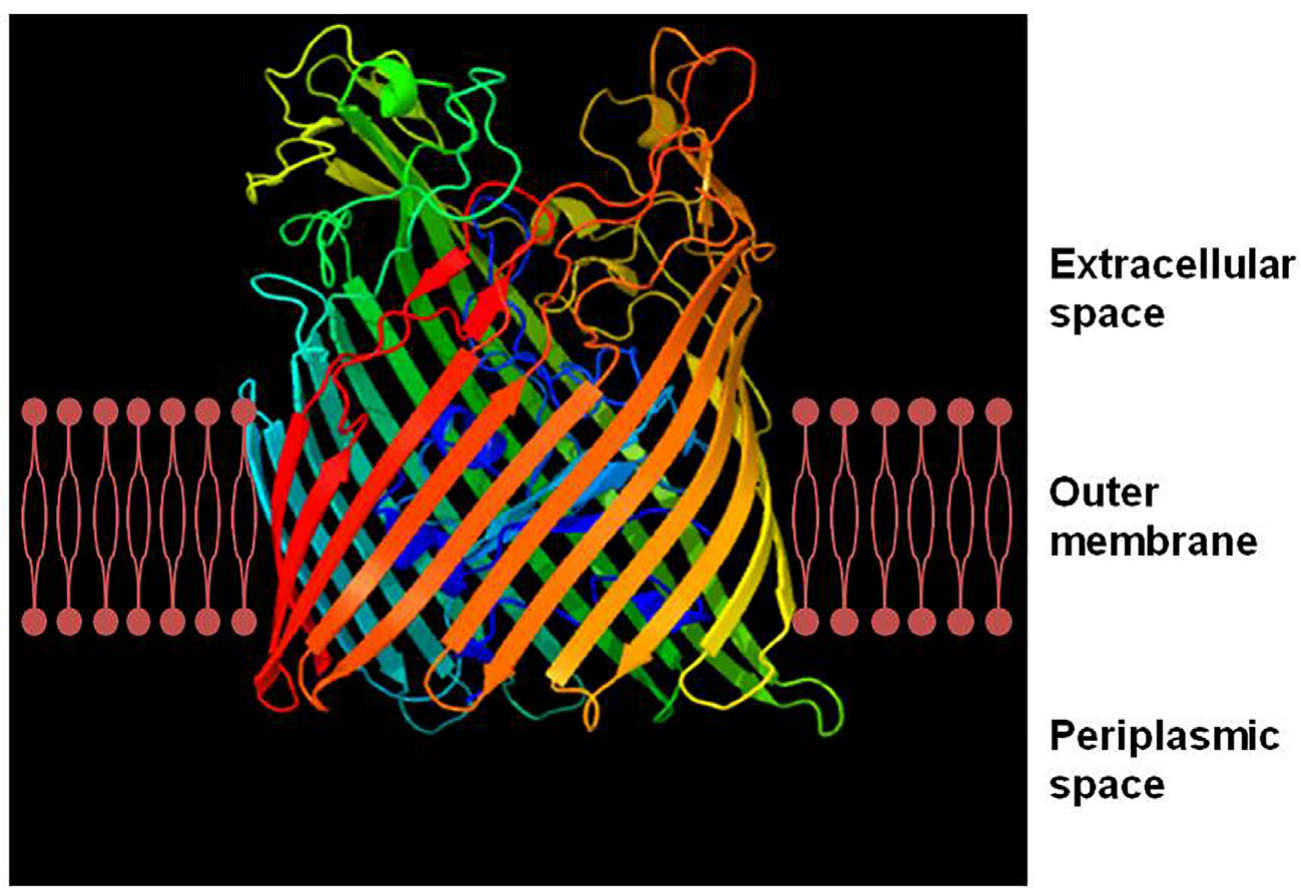
Ribbon diagram of FepA, a TonB-dependent transporter (TBDT) from *Escherichia coli*. TonB-dependent transporters possess two domains, a 22-stranded anti-parallel β–barrel spanning the outer membrane and an N-terminal globular plug domain that resides within the barrel. The β–strands of the barrel are connected on the extracellular side by long flexible loops and on the periplasmic side by short turns. The ribbon diagram of FepA was generated with the Phyre2 software (Kelley et al., 2015).

**Figure 2:**
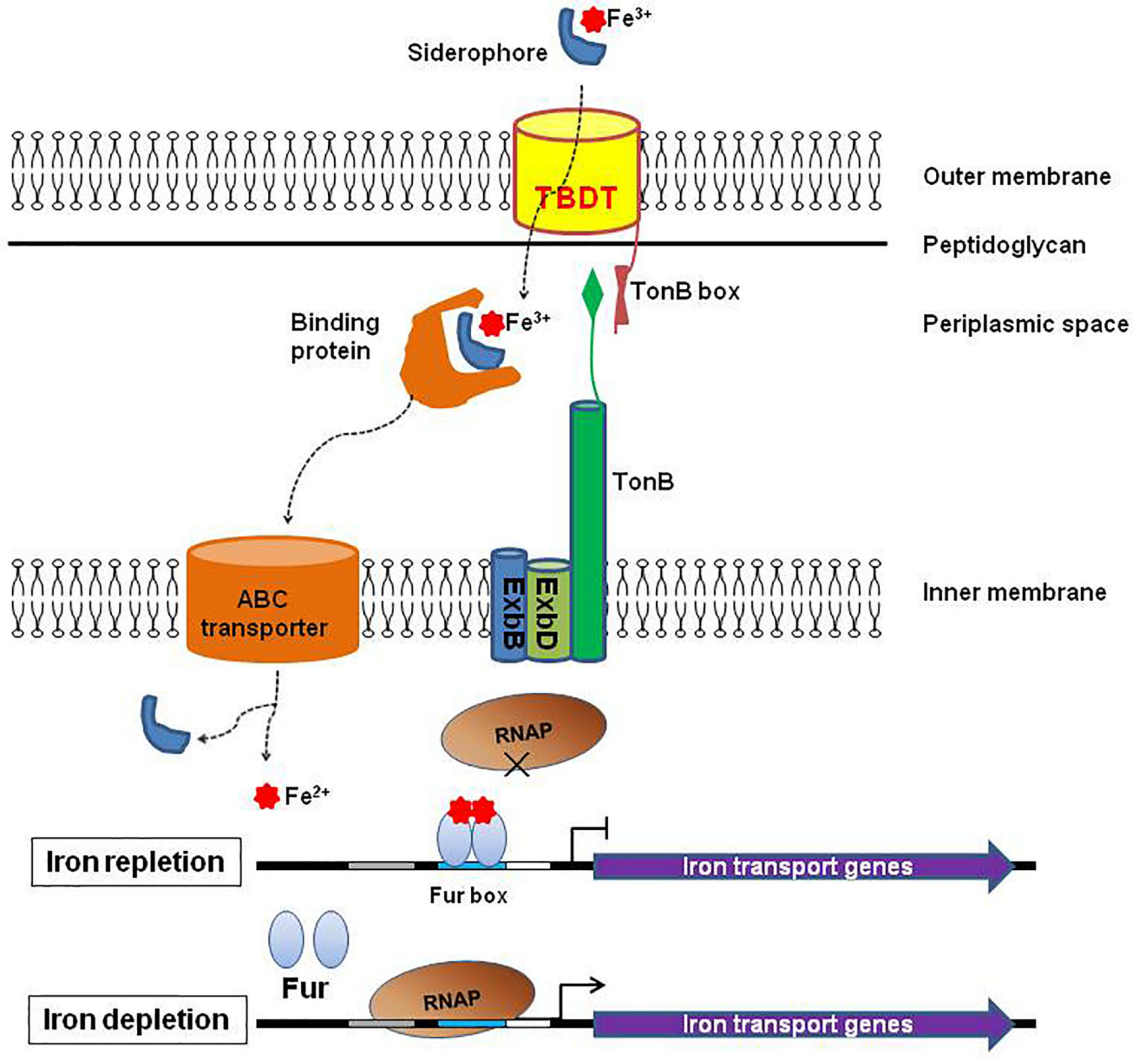
Diagram of substrate transport *via* TonB-dependent transporter (TBDT). Siderophore secreted by Gram-negative bacteria chelates ferric ion (Fe^3+^) from Fe^3+^–containing compounds in the extracellular environment. TonB-dependent transporter (TBDT) then mediates transport of ferric siderophore through the outer membrane into the periplasmic space. This process is driven by energy from an inner membrane protein complex, TonB-ExbB-ExbD. In the periplasm, the ferric siderophore is taken up by a binding protein and delivered through the inner membrane by an ABC-type transport complex. There the ferric siderophore is hydrolyzed, and then reduced to ferrous ion (Fe^2+^) by enzymes to supply iron for cell metabolism. Expression of iron-transport genes (including TBDT-encoding genes) is under the strict regulation of the ferric uptake regulator (Fur). Fur binds the Fur box sequence within the promoter region of the target genes using excess Fe^2+^ as a cofactor, and blocks the access of RNA polymerase leading to the repression of the target genes. However, when Fe^2+^ is depleted, Fur cannot bind the Fur box resulting in depression of the target genes.

All TBDTs are structurally similar (Noinaj et al., 2010). The structure of TBDT comprises two domains: a 22-stranded β-barrel spanning the outer membrane and a globular plug domain folded into the barrel interior. The β-barrel domain forms a transport channel for the substrate. The β–strands are connected sequentially with long loops on the extracellular side of the barrel and short turns on the periplasmic side (**Figure 1**). The extracellular loops are required for substrate binding and transport through the TBDT protein (Pienko and Trylska, 2020). The plug domain contains a consensus sequence, TonB-box, at the N terminus (Noinaj et al., 2010; Krewulak and Vogel, 2011). The plug domain binds the substrate at the extracellular side of the outer membrane, and its periplasmic region (particularly, the TonB-box) interacts with the TonB-ExbB-ExbD complex to tap energy derived from the proton motive force across the inner membrane, which is required for the substrate transport. The substrate in the periplasmic space is subsequently transferred into the cytoplasm through an ABC transporter in the inner membrane (**Figure 2**) (Noinaj et al., 2010; Krewulak and Vogel, 2011; Gresock and Postle, 2017). Siderophores are the most common transport substrates of TBDTs. However, recent studies have revealed the diversity of TBDT substrates, including vitamin B12, nickel chelates, zinc chelates, carbohydrates, and enzymes (Schauer et al., 2008).

## Characteristics of TBDTs Make Them Excellent Candidates for the Vaccine Development of Gram-Negative Bacteria

An ideal target for the vaccine development should have the following features: (i) surface exposure, (ii) wide distribution in pathogenic strains, (iii) capable of eliciting protective immune responses, (iv) inducible expression during infection, and (v) plays a critical role in pathogenesis (Wieser et al., 2010).

The characteristics of TBDTs meet most standards of the ideal target for the vaccine development. As outer membrane proteins, TBDTs have many surface-exposed epitopes, which are targets of the host immune system (Solis and Cordwell, 2011; Zeng et al., 2017). For example, the extracellular loops that connect the β–strands of TbpA, a TBDT essential for the uptake of transferrin-iron in *Neisseria gonorrhoeae*, are surface-exposed and immunogenic. The specific antibodies against these loops can prevent the ligand binding capabilities of TbpA, therefore, inhibit bacterial growth *in vivo* (Boulton et al., 2000; Cornelissen et al., 2000; Masri and Cornelissen, 2002). Moreover, the specific antibodies can bind to the TBDT epitopes of *Acinetobacter baumannii* and form immune complexes, which can be recognized by phagocytes and facilitate bacterial elimination (Goel and Kapil, 2001).

Due to the important roles of substrates in bacterial survival in the host, TBDTs are required for the pathogenicity of various pathogens, including *Salmonella* *enterica* (Kingsley et al., 1999; Williams et al., 2006; Hu et al., 2009; Xiong et al., 2012), *Francisella tularensis* (Sen et al., 2010), *Pseudomonas* *fluorescens* (Sun and Sun, 2015), *Haemophilus* *ducreyi* (Leduc et al., 2008), *Riemerella anatipestifer* (Lu et al., 2013), *Proteus mirabilis*  *(* Nielubowicz et al., 2008), *Pseudomonas aeruginosa* (Mastropasqua et al., 2017), *Neisseria meningitidis* (Kumar et al., 2012), et al. For example, The catecholate siderophores have a strong affinity for iron (Wandersman and Delepelaire, 2004), and play a key role in the iron uptake and *in vivo* growth of *Salmonella* *enterica* (Williams et al., 2006; Wellawa et al., 2020). Transport of these catecholate siderophores is dependent on three TBDTs, IroN, FepA, and CirA. Therefore, deficiency of these transporters of catecholate siderophores led to significant virulence reduction in *S*. Typhimurium (Wellawa et al., 2020).

The TBDTs proteins are highly regulated at transcriptional and posttranscriptional levels; and the virulence-associated TBDTs are generally induced *in vivo* (Hagan and Mobley, 2007; Noinaj et al., 2010). For example, the ferric uptake regulator (Fur) plays a key role in the regulation of TBDTs for siderophore (Sun and Sun, 2015; Becerra et al., 2020). In the presence of iron, Fur binds a specific 19 bp DNA sequence, called the “Fur box”, using Fe^2+^ as a cofactor within the promoter region of the regulated genes. The binding of Fur prevents the access of RNA polymerase leading to the repression of the downstream genes. However, under iron limiting conditions (for example, *in vivo*), Fur cannot bind the sequence of the Fur box resulting in derepression of genes that encode siderophore transporters (including TBDTs), proteins involved in siderophore biosynthesis and iron metabolism (**Figure 2**) (Lee and Helmann, 2007; Noinaj et al., 2010). In addition to Fur, multiple regulatory factors including small RNAs, ChvR (Frohlich et al., 2018), OmrA and OmrB (Guillier and Gottesman, 2008), string response regulators (P) ppGpp and DksA (Zhang et al., 2019), participate in the regulation of TBDTs for siderophore.

## Application of TBDTs in the Vaccine Development of Gram-Negative Bacteria

### Attenuated Vaccine

Because many of the substrates transported by TBDTs are essential for the survival and pathogenicity of pathogens *in vivo*, mutation of the encoding genes of these TBDTs will lead to significant decrease of bacterial virulence. Therefore, some TDBT-encoding genes are suitable targets for the construction of attenuated vaccine strains (**Table 1**).

**Table 1:** Application of TonB-dependent transporters (TBDTs) in vaccine development of Gram-negative bacteria.

Vaccine candidates	Results	References
**Attenuated vaccine**		
*Salmonella* *enterica* the *fepA iroN cir* triple mutant	The mutant was significantly attenuated in a mouse model. No immunized mice were killed by the i.g. or i.v. challenges at lethal doses.	(Williams et al., 2006)
the *foxA* mutant	The LD_50_s of the mutant (>10^9^ CFU i.g.; >10^4^ CFU i.v.) were significantly higher than those of the wild-type (10^5^ CFU i.g.; <10 CFU i.v.). Immunization of the mutant significantly protected the immunized mice from the intragastric challenge of the wild-type.	(Kingsley et al., 1999)
Δ*yncD*	The Δ*yncD* mutant is 1000 times less virulent than the wild-type in the mouse mucin model. Intranasal inoculation of Δ*yncD* showed a significant immunoprotection against the lethal intraperitoneal challenge of the wild-type.	(Xiong et al., 2012)
*Shigella dysenteriae* Δ*icsA*Δ*ent*Δ*fep*Δ*stxA*	Clinical trials showed that the vaccine was well tolerated (the maximum tolerable dose >10^8^ CFU). A single oral vaccination induced a significant circulating IgA ASC and serum antibody response.	(Sadorge et al., 2008; Launay et al., 2009)
*Riemerella anatipestifer* Δ*B739_1343*	The LD_50_ of the mutant was increased by >1000 times compared to that of the wild-type in a ducking model. Intramuscular immunization with Δ*B739_1343* conferred a protection rate of 83.33% in the immunized ducks group against the lethal challenge.	(Liu et al., 2018)
**Subunit vaccine**		
Uropathogenic *Escherichia coli* IreA, Hma, IutA, FyuA	Intranasal immunization of mice with the four TBDTs stimulates a protective systemic and mucosal immune responses that correlate with significant reductions in bladder and/or kidney bacterial load and significant protection from experimental infection.	(Alteri et al., 2009; Serino et al., 2010; Brumbaugh et al., 2013; Mobley and Alteri, 2015; Habibi et al., 2017)
*Pseudomonas* *aeruginosa* FpvA	Intranasal peptide-based FpvA-KLH conjugate vaccine of mice elicited production of IgG and IgM antibodies in sera, IgA antibodies in lung supernatant and antigen-specific IL-17. FpvA-KLH immunization significantly reduced bacterial burden and pulmonary edema, protected mice from acute murine pneumonia of *P*. *aeruginosa*.	(Sen-Kilic et al., 2019)
*Neisseria meningitidis* TbpA	Subcutaneous inoculation of mice with recombinant TbpA conferred significant protection (100% at the challenge dose of 2×10^7^ CFU; 85% at the challenge dose of 2×10^8^ CFU) against the lethal challenges of *N*. *meningitidis*.	(West et al., 2001)
FetA (FrpB, HxuC)	FetA can induce bactericidal antibody, and is a vaccine component.	(Pettersson et al., 1990; Ala’Aldeen et al., 1994; Kortekaas et al., 2006)
ZnuD	ZnuD can elicit bactericidal antibodies upon immunization in mice.	(Stork et al., 2010)
*Acinetobacter* *baumannii* BauA, BfnH	Subcutaneous immunization of mice with BauA and BfnH conferred survival rates of 45% and 40%, respectively, against the lethal challenge of *A*. *baumannii*. Passive immunization resulted in approximately 50% of survival rate in the groups receiving anti-BfnH or a combination of anti-BfnH and anti-BauA.	(Aghajani et al., 2019)
Extraintestinal pathogenic *Escherichia coli* IutA, IroN	Immunization of mice with recombinant IutA or IroN elicited high liters of total IgG antibody of IgG1/IgG2a isotypes at day 40 post-immunization, leading to protection against the lethal and non-lethal sepsis challenges.	(Mellata et al., 2016)
FyuA, IroN, IutA, ChuA	Nasal administration of two artificial chimeric polypeptides comprising the immunogenic epitopes of FyuA, IroN, IutA, ChuA, and UPEC-specific protein UspA stimulated strong humoral, cellular, and mucosal immune responses, and conferred protection with a significant reduction of bacterial load in a mouse model of ExPEC peritonitis.	(Wieser et al., 2010)
*Campylobacter jejuni* CfrA	CfrA-specific IgG blocked the ligand binding site of CfrA and consequently inhibited enterobactin-mediated growth promotion under iron-restricted conditions. The inhibitory effect was dose dependent.	(Zeng et al., 2009)
*Salmonella* *enterica* IroN	Intramuscular immunization of chicken with IroN elicited a survival rate of 90% against the intravenous challenge of *S*. Enteritidis relative to the 20% survival rate of the control group.	(Kaneshige et al., 2009)
*Neisseria gonorrhoeae* TbpA	Vaccine formulations comprising epitopes of TbpAB and inactivated cholera toxin induced potentially protective antibodies.	(Cornelissen, 2008)
*Klebsiella pneumonia* Kleb-SRP	Kleb-SRP was prepared by purifying siderophore receptor and porin proteins from *K*. *pneumonia*. Administration of cows with the vaccine before calving significantly decreased the risk of *Klebsiella* and total coliform mastitis by 76.9% and 47.5% respectively.	(Gorden et al., 2018)
*Aeromonas hydrophila* Tdr	Immunization with recombinant Tdr protein emulsified with non-mineral oil adjuvant protected Catfish fingerlings against the challenge of the virulent *A*. *hydrophila* strain with a relative survival rate of 95.59%. The bacterial burdens in the liver, spleen, and anterior kidney of the immunized catfish were also markedly reduced.	(Abdelhamed et al., 2017)
*Pseudomonas* *fluorescens* TdrA	Immunization with recombinant TdrA protected the immunized flounders against the lethal challenge of *P*. *fluorescens* with a relative percent of survival of 80.6%.	(Hu et al., 2012)
TfeR	Immunization of turbot with recombinant TfeR induced high titers of specific serum antibodies, which bound the *P*. *fluorescens* cells, and greatly reduced bacterial infectivity to fish cells. The relative percent of survival of the TfeR immunization was 50%.	(Sun and Sun, 2015)
*Haemophilus ducreyi* HgbA	Anti-HgbA IgG blocked hemoglobin binding to the HgbA receptor. Immunization with HgbA reduced initial size and severity of lesion formation and/or increased the speed of healing in a swine model of chancroid infection. Immunized pigs effectively eliminated pathogens at the inoculation site. Passive immunization also protected the pigs from the homologous challenge.	(Afonina et al., 2006; Nepluev et al., 2009; Leduc et al., 2011)
*Haemophilus parasuis* HxuC	Vaccination of mice with HxuC elicited significant humoral immune responses and increased the levels of IL-2, IL-4, IFN-γ. The HxuC immunization alleviated the tissue damage levels in the mouse model of intraperitoneal infection of *H*. *parasuis* and conferred protection against the lethal challenge with a survival rate of 87.5%.	(Wen et al., 2016)
FhuA	FhuA was immunogenic and elicited specific antibodies during the course of natural infection.	(del Rio et al., 2006)
*Pasteurella multocida* TbpA	Subcutaneous immunization with recombinant TbpA fragments with varied lengths stimulated high levels of total IgG and subtypes IgG1 and IgG2a in the mice serum. The immunization protected the immunized mice against lethal challenges with survival rates from 16.7%–50%.	(Shivachandra et al., 2015)

LD_50_, median lethal dose; i.g., intragastrically; i.v., intravenously.

#### Salmonella enterica

Three TBDTs are available for the transport of catecholate siderophore in *Salmonella enterica*. Cir transports 2, 3-dihydroxybenzoylserine; FepA facilitates the uptake of enterobactin and 2, 3-dihydroxybenzoylserine; IroN is the receptor for salmochelins, enterobactin, and 2, 3-dihydroxybenzoylserine. Intragastric and intravenous immunization of the triple deletion mutant elicited significant protective immune responses against the wild-type challenge by either route in the mouse model (Williams et al., 2006).

FoxA mediates the transport of ferrioxamines across the outer membrane. A *foxA* mutation substantially reduced bacterial colonization in rabbit ileal loops, resulting in virulence reduction. Furthermore, immunization of the *foxA* mutant protected mice against the wild-type challenges (Kingsley et al., 1999).

YncD is a putative TBDT of *S*. Typhi, which had been identified as an *in vivo*-induced antigen probed with sera from Typhoid fever patients (Hu et al., 2009). Nasal inoculation of the *yncD*-deleted mutant elicited a significant immunoprotection against the lethal wild-type challenge (Xiong et al., 2012). The *yncD* gene was also used in the construction of the attenuated vaccine strains of *S*. Paratyphi A (Xiong et al., 2015; Zhu et al., 2015).

#### Shigella dysenteriae

In some cases, TBDT-encoding genes were used as one of the target genes in strain construction. For example, the SC599 vaccine is an attenuated strain of *S*. *dysenteriae* with the deletion of four genes. The *fep* gene that encodes a TBDT for enterobactin, was deleted in addition to the mutation of three other virulence genes, *icsA* (essential for bacterial spreading), *ent* (encoding enterobactin synthase), and *stxA* (encoding the shiga toxin active A subunit) to construct the strain. Clinical trials revealed that the SC599 vaccine was well tolerated. A single oral vaccination elicited significant IgA ASCs and antibody responses (Sadorge et al., 2008; Launay et al., 2009).

#### Riemerella anatipestifer

*R*. *anatipestifer*, which causes acute septicemia and infectious polymastitis in ducks, chickens, geese, and other avian species, is a serious threat to the poultry industry. Liu et al. demonstrated that a putative TonB-dependent iron transporter of *R*. *anatipestifer* CH-1, namely, B739_1343, played an important role in iron acquisition and bacterial pathogenicity. The deletion of B739_1343 led to a significant decrease in virulence. Intramuscular immunization with the deletion mutant protected 83.33% of the immunized ducks against intramuscular challenges of the wild-type strain at a high dose of 100-fold LD_50_ (Liu et al., 2018).

### Subunit Vaccine

TBDTs comprise a large part of the identified proteins with vaccine potential in large-scale screens based on genomics, proteomics, reverse and structural vaccinology. A total of 18 β-barrel transmembrane proteins and 8 outer membrane lipoproteins were identified in an effort to discover novel leptospiral vaccine candidates. Many β-barrel transmembrane proteins (6/18) are TonB-dependent receptors associated with nutrient transportation (Grassmann et al., 2017). In another effort to determine potential core vaccine targets of *A*. *baumannii*, two TBDTs, namely FhuE receptor and HMPREF0010_01517, are identified (Hassan et al., 2016). Researchers used a large-scale selection process combined with bioinformatic, genomic, transcriptomic, and proteomic in a screen of vaccine candidate proteins for uropathogenic *Escherichia coli*. Six suitable vaccine candidates, including ChuA, Hma, Iha, IreA, IroN, and IutA, were screened from the 5379 predicted proteins. All these candidates are TBDTs involved in iron uptake (Alteri et al., 2009). So far many TBDTs from various pathogens had been evaluated their potential as subunit vaccines. The important progresses are summarized in **Table 1** and described in the following sections.

#### Uropathogenic *Escherichia coli* (UPEC)

UPEC is among the most prevalent agents of urinary tract infections. Four TBDTs (IreA, Hma, IutA, and FyuA) associated with iron acquisition had been identified in systematic screens. Nasal immunization of each antigen, with cholera toxin as adjuvant, protected experimentally infected mice from colonization of the bladder and/or kidneys by UPEC (Alteri et al., 2009; Serino et al., 2010; Brumbaugh et al., 2013; Habibi et al., 2017). The four antigens can be combined to produce a multi-subunit vaccine (Mobley and Alteri, 2015).

#### Pseudomonas aeruginosa

*P*. *aeruginosa* is one of the leading opportunistic pathogens responsible for acute and chronic respiratory tract infections with high morbidity and mortality. An *in vivo*-induced ferripyoverdine transporter, FpvA, is essential for virulence of *P*. *aeruginosa*. A subunit vaccine comprising the extracellular peptides of FpvA conjugated to keyhole limpet hemocyanin (KLH) was produced to evaluate its immunoprotective efficacy in a murine model. Nasal vaccination of FpvA-KLH elicited IgG and IgM antibodies in sera and IgA antibodies in lung supernatant. CD11b^+^ dendritic cells and memory CD4^+^ T cells were induced in the lungs of immunized mice. Furthermore, FpvA-KLH immunization significantly reduced bacterial burden and pulmonary edema (Sen-Kilic et al., 2019).

#### Neisseria meningitidis

*N*. *meningitidis* is the etiologic agent of meningococcal meningitis, which is a serious public health issue in developed and developing countries. *Neisseria* cells steal iron from human iron-binding proteins, such as hemoglobin, transferrin, and lactoferrin, for survival. The immunoprotection of recombinant transferrin binding proteins, TbpA (a TBDT) and TbpB, had been evaluated against meningococcal infection in a mouse model. TbpA was demonstrated to confer protection against the challenge of *N*. *meningitidis* and had cross-protection against the isolates of serogroup B and serogroup C (West et al., 2001; Noinaj et al., 2012; Noinaj et al., 2013).

FetA (also known as FrpB, HxuC) is an outer membrane heme transporter of *N*. *meningitidis* (Saleem et al., 2012). FetA has been demonstrated to induce bactericidal antibody and regarded as a vaccine component (Pettersson et al., 1990; Ala’Aldeen et al., 1994; Kortekaas et al., 2006). However, the antibody is strain-specific due to the high variations of surface-exposed fragments (Thompson et al., 2003).

In addition to TonB-dependent iron transporters, an outer membrane zinc transporter, ZnuD, is also required for bacterial survival and plays a critical role in the pathogenicity of *N*. *meningitidis*. ZnuD, which is highly conserved in the isolates of *N*. *meningitidis*, can elicit bactericidal antibodies upon immunization in mice (Stork et al., 2010).

#### Acinetobacter baumannii

*A*. *baumannii* is an opportunistic pathogen that accounts for serious life-threatening nosocomial infections due to its easy acquisition of antibiotic resistance and its persistence in the environment (Greene et al., 2016). BauA and BfnH are two TBDTs in *A*. *baumannii* that facilitate the uptake of siderophores from the acinetobactin cluster and cluster I, respectively (Eijkelkamp et al., 2011). Both TBDTs were identified as vaccine candidates using an assay of reverse vaccinology (Ni et al., 2017). The whole proteins of BauA and BfnH showed partial protective effects in animal experiments. Death rate and bacterial burdens in the main organs of immunized mice were decreased compared with those of the control group. Passive immunization resulted in approximately 50% of survival rate in the immunized groups of BfnH or a combination of BfnH and BauA (Aghajani et al., 2019).

#### Extraintestinal Pathogenic *Escherichia coli* (ExPEC)

ExPEC reside in the intestine as members of normal flora. However, ExPEC cells cause infections when entering extraintestinal sites, such as blood, urinary tract, and meninge, due to their specific virulence factors. ExPEC are the leading cause of community and nosocomial bacterial sepsis with a mortality rate of 30%–50%. Salmochelin and aerobactin mediate the iron uptake of ExPEC, which is essential for bacterial virulence (Garenaux et al., 2011). Their corresponding transporters, IroN and IutA, are widely distributed in human isolates of ExPEC (Mellata et al., 2009).

Immunogenicity of IroN was first evaluated by Russo et al. (2003). Subcutaneous inoculation of recombinant IroN induced a significant increase in anti-IroN IgG in immunized mice, leading to a significant reduction of bacterial burdens in the kidneys but not the bladders of the mice challenged with ExPEC. Subcutaneous immunization of mice with recombinant IutA and IroN elicited strong humoral immune responses and protected immunized mice against the lethal and non-lethal sepsis challenges. Passive immunization also reduced bacterial burdens in organs and blood of the tested mice (Mellata et al., 2016).

Wieser et al. in silico predicted immunogenic epitopes of the extracellular loops of the *E. coli* outer membrane siderophore receptor FyuA, IroN, and IutA, the heme/hemoglobin TonB-dependent receptor ChuA, and the uropathogenic *E. coli* (UPEC)-specific protein UspA, and combined them into two artificial chimeric polypeptides. Nasal administration of the two novel multi-epitope subunit vaccines elicited strong humoral and cellular immune responses, and conferred a high degree of protection in a mouse model of ExPEC peritonitis (Wieser et al., 2010).

#### Campylobacter jejuni

*Campylobacter* is one of the most common bacterial causes of human enteritis in many industrialized countries (Ketley, 1997). Zeng et al. found that a ferric enterobactin receptor, CfrA, was dramatically induced under iron-restricted conditions and during infections. The anti-CfrA serum blocked the function of iron uptake and diminished ferric enterobactin-mediated growth promotion under iron-restricted conditions (Zeng et al., 2009).

#### Salmonella enterica

Vaccination is one of the efficient means to control the contamination of *Salmonella* Enteritidis in poultry farming. Kaneshige et al. purified natural IroN from *S*. Typhimurium using affinity chromatography. Intramuscular immunization of chicken with purified natural IroN conferred a survival rate of 90% against the intravenous challenge of *S*. Enteritidis compared with the 20% survival rate of the control group (Kaneshige et al., 2009). The data showed that IroN is an important protective antigen against *Salmonella* infection in chickens.

#### Neisseria gonorrhoeae

*N*. *gonorrhoeae* causes gonorrhea, a global public health issue with an estimated incidence of 62.4 million cases each year. The transferrin iron transport system, TbpAB, is not subject to antigenic variation and is widely distributed in *Neisseria* isolates. Vaccine formulations comprising epitopes of TbpAB elicited potentially protective antibodies (Cornelissen, 2008).

#### Klebsiella pneumonia

Mastitis caused by *Klebsiella* spp. is an emerging issue in the dairy industry. A *K*. *pneumoniae* vaccine (Kleb-SRP) was prepared by purifying siderophore receptor and porin proteins from fermentation cultures of *K*. *pneumonia*. Subcutaneous vaccination of cows with Kleb-SRP before calving decreased the risk of *Klebsiella* and total coliform mastitis by 76.9% and 47.5%, respectively (Gorden et al., 2018).

#### Aeromonas hydrophila

A TonB dependent transporter, Tdr, presents in the virulent strains of *A*. *hydrophila* but was absent in strains of low virulence. Immunization with Tdr induced significant antibody responses and protected immunized catfish fingerlings against the challenge of the virulent strain, and the relative survival rate was 95.59%. The bacterial burdens in the organs of immunized catfish were also markedly reduced (Abdelhamed et al., 2017).

#### Pseudomonas fluorescens

*P*. *fluorescens* is a common pathogen of aquaculture. The purified recombinant TdrA, a TonB-dependent outer membrane transporter, is a promising subunit vaccine. Immunization of recombinant TdrA protected the immunized flounders effectively against the lethal challenge of *P*. *fluorescens* with a relative percent of survival of 80.6% (Hu et al., 2012).

A ferric enterobactin transporter of *P*. *fluorescens*, TfeR, is essential for bacterial virulence in turbot. Inoculation of recombinant TfeR induces specific serum antibodies and displays significant protection against the challenge of pathogenic strain. The TfeR antibodies bind with bacteria and block their infection (Sun and Sun, 2015).

#### Haemophilus ducreyi

*H*. *ducreyi* is the cause of chancroid. A hemoglobin transporter, HgbA, promotes heme acquisition and is required for the survival and pathogenicity of *H*. *ducreyi*. Immunization of purified native HgbA prevented the development of chancroid in a swine model. Immunized animals effectively eliminated pathogens at the inoculation site. Anti-HgbA IgG blocks the binding of hemoglobin to HgbA and restricts the capability of *H*. *ducreyi* to acquire heme/iron and then promotes the elimination of pathogens (Afonina et al., 2006; Nepluev et al., 2009; Leduc et al., 2011).

#### Haemophilus parasuis

*H*. *parasuis* is the etiological agent of Glasser’s disease in pigs. The *hxuC* gene encodes a transporter for heme/hemopexin utilization and is required for bacterial virulence (Morton et al., 2007). Wen et al. found that immunization of HxuC induced strong immune responses and protected immunized mice against the lethal challenge of the virulent strain with 87.5% survival rate. The antiserum inhibited the bacterial growth of *H*. *parasuis* (Wen et al., 2016). FhuA is involved in ferric hydroxamate uptake of *H*. *influenzae*. Animal experiments showed that FhuA was immunogenic and induced specific antibodies during porcine infection (del Rio et al., 2006).

#### Pasteurella multocida

Hemorrhagic septicemia is a serious disease of cattle and buffalo mainly caused by the pathogenic serogroups of *P. multocida* (Shivachandra et al., 2015). Transferrin-binding protein A (TbpA) is an iron acquisition TBDT prevalent in *P. multocida*. Shivachandra et al. evaluated the immunogenicity of recombinant TbpA fragments with varied lengths. Immunization with TbpA fragments induced high levels of IgG, IgG1, and IgG2a in the mice serum and protected the immunized mice against lethal challenges with survival rates ranging from 16.7% –50% (Shivachandra et al., 2015).

## Advantages and Disadvantages of TBDTs in the Vaccine Development

Numerous studies have shown that TBDTs conferred significant immune protection against infection caused by their host bacteria. The characteristics revealed in these studies make TBDTs excellent candidates for the vaccine development (**Table 2**). However, antigenic variation hinders the application of some TBDTs in the vaccine development (**Table 2**).

**Table 2:** Advantages and disadvantages of TonB-dependent transporters (TBDTs) in vaccine development.

Advantages	Disadvantages
1 Surface exposure 2 Inducible expression during infection 3 Essential for bacterial virulence 4 Wide distribution in Gram-negative bacteria 5 Eliciting protective immune responses 6 The DNA sequences of TBDTs have distinctive features, which can be in silico annotated.	1 Antigenic variation 2 Single TBDT antigen is sometimes insufficient for the vaccine development

Due to their surface location and key roles in virulence, TBDTs are subjected to high selection pressure, which results in frequent variations in some TBDTs. For example, vaccine efforts of *N. gonorrhoeae* have been hampered by the frequent antigenic variation of most surface structures. *N. gonorrhoeae* has two variation mechanisms. The first one is homologous recombination, which is employed by the pilin protein (Criss et al., 2005). The second mechanism is the slipped-strand mispairing mechanism responsible for the variable expression of some iron transport proteins, including LbpAB, HpuAB, and FetA. These proteins contain polymeric repeat regions in their encoding loci and promote a rapid on–off switching of protein expression (Bayliss et al., 2001; Harrison et al., 2013).

A single TBDT antigen may be insufficient to protect against bacterial infection because it is unlikely to have 100% epidemic coverage in the pathogenic strains due to its frequent variation (Sarkissian et al., 2019). Therefore, multiple TBDTs are occasionally required to prepare a qualified vaccine against infection.

Overall, a large number of TBDTs with transporting function of certain rare nutrients have been demonstrated to have application potential in the immune control of infection caused by Gram-negative bacteria. This large category of molecules will definitely play a non-negligible role in the future vaccine development.

## Author Contributions

Conceptualization, YC and WH. Writing—original draft preparation, JW, KX, and QP. Supervision, YC and WH. All authors contributed to the article and approved the submitted version.

## Funding

YC was supported by the National Natural Science Foundation of China under Grant number 81974299, the Department of Science and Technology of Sichuan province under Grant number 2019YJ0693, the Southwest Medical University—Traditional Medicine Hospital Affiliated to Southwest Medical University under Grant number 2018XYLH-017, and the Scientific Research Starting Foundation of Traditional Medicine Hospital Affiliated to Southwest Medical University (2019119). JW was supported by the Southwest Medical University—Traditional Medicine Hospital Affiliated to Southwest Medical University under Grant number 2020XYLH-078. KX was supported by the National Natural Science Foundation of China under Grant number 31500116.

## Conflict of Interest

The authors declare that the research was conducted in the absence of any commercial or financial relationships that could be construed as a potential conflict of interest.
